# AFLP analysis reveals high genetic diversity but low population structure in *Coccidioides posadasii* isolates from Mexico and Argentina

**DOI:** 10.1186/1471-2334-13-411

**Published:** 2013-09-03

**Authors:** Esperanza Duarte-Escalante, Gerardo Zúñiga, María Guadalupe Frías-De-León, Cristina Canteros, Laura Rosio Castañón-Olivares, María Rocío del Reyes-Montes

**Affiliations:** 1 Departamento de Microbiología y Parasitología, Facultad de Medicina, Universidad Nacional Autónoma de México (UNAM), Ciudad Universitaria No. 3000, México, D. F. 04510, México; 2 Departamento de Zoología, Escuela Nacional de Ciencias Biológicas, Instituto Politécnico Nacional, Prol. de Carpio y Plan de Ayala. Col. Sto. Tomás, 11340, México, D. F., México; 3 División de Investigación, Hospital Juárez de México, Edificio E. Av. Instituto Politécnico Nacional 5160, Col. Magdalena de las Salinas, 07760, México, D. F., México; 4 Departamento de Micología, INEI ANLIS “Dr. Carlos G. Malbrán”, Av. Velez Sarsfield 563, 1281, Buenos Aires, Argentina

**Keywords:** Genotypic variability, Haplotypes, Reproductive system

## Abstract

**Background:**

*Coccidioides immitis* and *C. posadasii* cause coccidioidomycosis, a disease that is endemic to North and South America, but for Central America, the incidence of coccidioidomycosis has not been clearly established. Several studies suggest genetic variability in these fungi; however, little definitive information has been discovered about the variability of *Coccidioides* fungi in Mexico (MX) and Argentina (AR). Thus, the goals for this work were to study 32 *Coccidioides* spp. isolates from MX and AR, identify the species of these *Coccidioides* spp. isolates, analyse their phenotypic variability, examine their genetic variability and investigate the *Coccidioides* reproductive system and its level of genetic differentiation.

**Methods:**

*Coccidioides* spp. isolates from MX and AR were taxonomically identified by phylogenetic inference analysis using partial sequences of the *Ag2/PRA* gene and their phenotypic characteristics analysed. The genetic variability, reproductive system and level of differentiation were estimated using AFLP markers. The level of genetic variability was assessed measuring the percentage of polymorphic loci, number of effective allele, expected heterocygosity and Index of Association (*I*_*A*_). The degree of genetic differentiation was determined by AMOVA. Genetic similarities among isolates were estimated using Jaccard index. The UPGMA was used to contsruct the corresponding dendrogram. Finally, a network of haplotypes was built to evaluate the genealogical relationships among AFLP haplotypes.

**Results:**

All isolates of *Coccidioides* spp. from MX and AR were identified as *C. posadasii*. No phenotypic variability was observed among the *C. posadasii* isolates from MX and AR. Analyses of genetic diversity and population structure were conducted using AFLP markers. Different estimators of genetic variability indicated that the *C. posadasii* isolates from MX and AR had high genetic variability. Furthermore, AMOVA, dendrogram and haplotype network showed a small genetic differentiation among the *C. posadasii* populations analysed from MX and AR. Additionally, the *I*_*A*_ calculated for the isolates suggested that the species has a recombinant reproductive system.

**Conclusions:**

No phenotypic variability was observed among the *C. posadasii* isolates from MX and AR. The high genetic variability observed in the isolates from MX and AR and the small genetic differentiation observed among the *C. posadasii* isolates analysed, suggest that this species could be distributed as a single genetic population in Latin America.

## Background

Species of the genus *Coccidioides*, primarily *Coccidioides immitis* and *C. posadasii*, cause coccidioidomycosis, which is a disease endemic to North and South America [1]. This mycosis is most prevalent in the Southwestern United States (US), Northern Mexico (MX), Central America and the foothills region of South America [2]. The habitat conditions that permit the development of the saprophytic phase of these fungi are sandy soils in arid areas where the annual rainfall is less than 500 mm [3-5].

Coccidioidomycosis infection in humans and other mammals is caused by the inhalation of arthroconidia. The illness begins with acute respiratory symptoms that are typically benign and vanish spontaneously; however, the disease can evolve into progressive clinical forms that spread to the skin and subcutaneous, visceral and skeletal tissues. These severe progressive forms cause high morbimortality and are commonly associated with immunocompromised patients [6].

Coccidioidomycosis is an emerging disease because increased infection rates have been recorded in recent years from MX [7] and Argentina (AR) [8]. In endemic areas in the US, more than 100,000 primary human infections by *Coccidioides* spp. are estimated each year; a considerable increase in the incidence of this disease has been noted in recent years, particularly in California and Arizona. The increased incidence of the disease has been associated with a rise in the migration of individuals who have not previously been exposed to the fungus into the endemic areas [9].

The current epidemiology for coccidioidomycosis in MX is unknown, because no prevalence studies have been conducted in most Mexican states since 1960. However, according to the information that is available, more than 1,500 cases of primary coccidioidomycosis and 15 cases of disseminated disease are estimated annually. It is important to note that this estimate was based on epidemiological studies prior to 1994; since 1995, there are no records of coccidioidomycosis incidence in MX because this infection was excluded from reports prepared for the national epidemiological registry [10]. This suggests that the disease may have developed much as it has in the US, where prevalence and incidence rates have soared since the early 1990s [11].

Epidemiology in AR is similar to that in MX: there are few existing studies, although in recent years, several epidemiological studies have been performed. These studies include an investigation conducted by Canteros *et al*. [8] that sought to identify areas of endemic mycoses in 10 rural communities from the Teuco-Bermejito interfluve, which is in the Chaco province. Although results from this study demonstrated that *Histoplasma capsulatum* was the principal agent of endemic mycoses, the researchers also indicated that the climatic conditions of the area are optimal for *Coccidioides* development and thus that the potential for *Coccidioides* infections in the area should not be dismissed. In another recent study conducted by Canteros *et al*. [12], the authors performed a comprehensive retrospective review of all documented coccidioidomycosis cases in AR between 1892 and 2009. This review demonstrated that between 2006 and 2009, the disease incidence in the Catamarca province increased from a historical rate of less than 0.5 cases per 100,000 inhabitants to 2 cases per 100,000 inhabitants, indicating that coccidioidomycosis is an emerging disease in this region.

Because of the increased incidence of this disease in North and South America, several studies in recent decades have sought to apply molecular techniques to better understand the taxonomy and population biology of *Coccidioides*. Numerous studies*,* principally in the US, have focused on the genetic variability of *C. immitis* isolates and concluded that this fungus has high genetic recombination and no genetic structure among fungal isolates; however, the recombination process has never been observed [13-17]. A recent study that supports the presence of recombination was conducted by Jewell *et al*. [18], who used microsatellites to determine that outbreaks of coccidioidomycosis in Arizona, US, were caused by a single fungal clone that was likely hypervirulent, possessed a high level of genetic variation and showed no dominant subtypes among its isolates. The absence of genetic structure among *C. immitis* isolates and the presence of cryptic sex in both species of this fungus led to an investigation of whether isolates of *C. posadasii*, which is the dominant species in Latin American countries, have the same behavior as isolates of *C. immitis*, which is the dominant species in the Southwestern US. To find evidence that would indicate whether there is an expansion of the fungus in MX and AR, this study aimed to determine the pheno- and genotypic variability and the population structure of two populations (MX and AR).

## Methods

### Isolates

Thirty two clinical isolates of *Coccidioides* spp. were used, 21 isolates from MX: one isolate (M0104) provided by Instituto Nacional de Neurología y Neurocirugía; 6 isolates (M1204, M1404, M1505, M2305, M2805 and M3005) provided by UMAE Hospital de Especialidades No. 71, Instituto Mexicano del Seguro Social; one isolate (M3905) provided by Instituto de Diagnóstico y Referencia Epidemiológicos, Secretaría de Salud; one isolate (M5406) provided by Clínica Derma Care; one isolate (M5708) provided by Hospital Central “Dr. Ignacio Morones Prieto”; 6 isolates (HU2, HU11, HU12, HU18, HU19 and HU24) and one reference strain (HU1) provided by Hospital Universitario, Universidad Autónoma de Nuevo León; three isolates (37.3, 5256 and SiFe) provided by Departamento de Infectología, Instituto Nacional de Ciencias Médicas y Nutrición “Salvador Zubirán” and one isolate (MA) provided by Instituto Nacional de Pediatría; and 11 isolates from AR (972579, 073089, 073094, 073129, 073130, 073131, 083376, 083377, 083380, 083381 and 083382), provided by the Instituto Nacional de Enfermedades Infecciosas, ANLIS “Dr. Carlos G. Malbrán” (Additional file 1). The isolates and strains were preserved at 4°C in flasks with sterile water and in tubes with mycobiotic agar (Bioxón, Mexico, MX) both with and without mineral oil. The isolates and reference strain studied were deposited in the *Coccidioides* spp. Collection of the Laboratorio de Micología Molecular, Departamento de Microbiología y Parasitología, Facultad de Medicina, Universidad Nacional Autónoma de México (UNAM).

### Biosafety

The culture and DNA extraction procedures for the isolates used in the present study were conducted in accordance with Biosafety Level 3 (BSL3) conditions.

### Monospore cultures

From each isolate grown on mycobiotic agar for 1–2 weeks at 30°C, a conidial suspension was prepared using 1 mL of phosphate buffer (pH 7.4) and 0.05% Tween 20 (PBST). This suspension was diluted (1:1000) and 50 µL of the dilution was grown on mycobiotic agar. The Petri dishes were incubated at 30°C and observed for colony growth. One colony was selected from each plate and grown on mycobiotic agar slants at 30°C. The monospore cultures were then preserved in sterile water at 4°C.

### Identification of the species of the *Coccidioides* spp. isolates

The DNA of each isolate was obtained following the procedure by Williams *et al*. [19] and Calderón *et al*. [20]. It was extracted and purified using the DNeasy Plant Mini kit (Qiagen GmbH, Hilden, GE). The mycelium was lysed using a FastPrep-24 instrument (MP Biomedicals, Solon, OH, US) by homogenisation through a pattern with four periods of 40 s each at a speed of 6 m/s and placement of the tube on ice for 5 min between each period. The DNA concentration was determined by spectrophotometry and confirmed by gel electrophoresis on a 1.0% agarose gel with ethidium bromide (10 μg/ml), using different concentrations of λ phage (Invitrogen, Carlsbad, CA, US). The DNA was stored at 4°C.

The oligonucleotides designed by Bialek *et al*. [21] were used for PCR. For the first PCR reaction, the reaction mixture comprised 10 ng of DNA in a 25 µL reaction volume containing 1X buffer, 200 μM dNTPs (Applied Biosystems, Inc., Foster City, CA, US), 2.5 mM MgCl_2_, 1.0 U of *Taq* polymerase (Applied Biosystems) and 100 pmol of each oligonucleotide, CoI (5′-GTACTATTAGGGAGGATAATCGTT-3′) and CoII (5′-GGTCTGAATGATCTGACGCA-3′). The following program was used for the PCR: one cycle at 94°C for 5 min, followed by 40 cycles of 94°C for 30 s, 50°C for 30 s and 72°C for 1 min with a final extension step at 72°C for 5 min. A second PCR reaction was then conducted. The reaction mixture for this second reaction comprised 2 μL of the product from the first PCR in a total reaction volume of 25 μL that was composed of buffer, 200 μM dNTPs (Applied Biosystems), 1.5 mM MgCl_2_, 1 U of *Taq* polymerase (Applied Biosystems) and 100 pmol of each oligonucleotide, CoIII (5′-ATCCCACCTTGCGCTGTATGTTCGA-3′) and CoIV (5′-GGAGACGGCTGGATTTTTTAACATG-3′). For the second PCR, the following program was used: one cycle at 94°C for 5 min followed by 40 cycles of 94°C for 30 s, 60°C for 30 s and 72°C for 1 min with a final extension step at 72°C for 5 min. The amplifications were conducted in an Esco Swift® Maxi™ thermocycler (ESCO, Hatboro, PA, US). The amplified products were then separated on 1.5% agarose gels in 0.5X TBE buffer at 100 V for 60 min. A 100-bp DNA ladder (Invitrogen) was used to determine the molecular sizes of the products. The gel was visualised using a Gel Doc XR (Bio-Rad, CA, US) image documentation system.

To confirm the identity of all isolates as *C. posadasii*, the 526-bp amplicons obtained from the first PCR were purified using the QIAquick PCR kit (Qiagen) and sequenced at the Unidad de Biología Molecular, Instituto de Fisiología Celular, UNAM using an automated ABI Prism 3100 (Applied Biosystems) sequencer. The sequences were edited using Chromas Lite 2.3 software (http://www.technelysium.com.au/chromas.html) and the sequence alignments were analised by the BLAST algorithm [22] to check similarities among all fungal sequences deposited in the GenBank database.

### Phenotypic variation

#### Macromorphology

The 32 *C. posadasii* isolates grown on mycobiotic agar at 30°C for 10 days were observed to identify the morphological characteristics of each culture (colony colour and texture).

#### Growth rates

To determine the growth rates of the isolates, each isolate was grown on Petri dishes containing YEG-agar (1% yeast extract, 1% glucose and 1.5% agar) (Bioxón) for 10 days. Circles 7 mm in diameter were cut from the colony margins in each plate. Each circle was placed in the centre of a Petri dish with YEG-agar containing one of the following different NaCl concentrations: 0 M (0%), 0.034 M (2%), 0.068 M (4%), and 0.102 M (6%). The Petri dishes were then immediately incubated at 30°C. Growth was determined by measuring the diameters of the colonies (cm) after 4, 8, 10 and 15 days of incubation. Each experiment was repeated four times. Subsequently, the growth rate for each isolate was determined using the following formula: growth rate = [(diameter of the colony – diameter of inoculum)/15 days of incubation].

#### Conidial size

The diameters of 40 conidia from each isolate grown on mycobiotic agar (Bioxón) and incubated at 37°C for 10 days were measured using a calibrated ocular micrometre (Olympus America Inc., Melville, NY, US).

#### Phenotypic data analysis

The MATLAB ver. 6.1 software package (Mathworks, Inc., Natick, MA, US) was used to analyse the phenotypic data for the growth rates. A two-way factorial design with repeated measurements was used to evaluate the incubation times (in days) and the different NaCl concentrations in the growth media to determine whether the isolate growth rates differed. In addition, the isolates were analysed by comparing their various growth rates and classifying the samples by their countries of origin. To analyse the conidial size, means were compared using the Tukey test.

### Genotypic variability

#### Amplified Fragment Length Polymorphism (AFLP) assessments

The AFLP analyses were performed with DNA from the monospore cultures in accordance with Vos *et al*. [23] and Duarte-Escalante *et al*. [24]. The DNA was restriction digested with the endonucleases *Eco*RI and *Mse*I. After digestion, adaptors were ligated to the resulting fragments. The fragments were then preamplified using primers E (5′-GACTGCGTACCAATTC-3′) and M (5′-GACGATGAGTCCTGGTAA-3′). Following this preamplification, selective PCR was performed in which the selective primers were nearly identical to primer E or M but were extended by specific two- or three-nucleotide combinations at their 3′ terminus. Six primer combinations were used: E+AA:M+CAC, E+AA:M+CAT, E+AA:M+CTG, E+AA:M+CTC, E+AC:M+CAT, and E+AC:M+CTC. Primer labeling was performed by phosphorylating the 5′ end of the *Eco* RI primers with [γ -^32^P] ATP and T4 polynucleotide kinase and the amplified materials were analysed on 5% polyacrylamide slab gels. A 50-bp DNA ladder (Invitrogen) was used as a reference.

### Statistical analyses of AFLP results

Bands on different gels between 100 and 400 bp were analysed. The AFLP markers were visually recorded, compared with the 50-bp DNA marker ladder, manually coded and translated into binary data that indicated either their presence (1) or absence (0) (Additional file 2). From this AFLP marker data, estimates of the genetic diversity of the MX and AR isolates were calculated using the Shannon Index (S), assuming that each genotypic marker represented a distinct locus [25], and Nei’s measure of genetic diversity (h), calculated using allelic frequencies [26]. Additionally, an expected heterozygosity by population (H) and an average heterozygosis (Hw) were calculated using allele frequencies in accordance with the Bayesian method employed by Zhivotovsky [27]. An AMOVA analysis using FMAD v1.1β software [28] was used to calculate the partitioning of the molecular variance of the *C. posadasii* isolates from MX and AR at one hierarchical level (geographic origin).

The statistical significance of *Fst* and the partitioned molecular variance were evaluated by performing 10,000 random data permutations [29]. The genetic similarity between isolates was calculated with the Jaccard index. The genetic relationships among isolates were assessed by mean of the Unweighted Pair Group Method with Arithmetic Mean (UPGMA) using the Jaccard matrix. Distortion of the inferred tree was assessed with the cophenetic correlation coefficient (CCCr) which was calculated using the Mantel test [30]. Multivariate statistical methods were carried out using the NTSYS-PC program (version 2.0, Exeter software) [31]. In addition, a haplotype network was performed to evaluate the genealogical relationships among the AFLP haplotypes using the median-joining method [32] implemented in NETWORK 4.2.0.1 (http://www.fluxus-engineering.com/sharenet.htm) [33], where branches with different lengths represent levels of evolutionary change. The parameters used were ϵ = 0, 1:1 weight for transitions-transversions and connection criteria. To distinguish clonal and recombinant structures in *C. posadasii*, the Index of Association (*I*_*A*_), was used, which is a statistical test that measures the degree of non-random association between alleles at different loci (linkage disequilibrium) [34]. Therefore, *I*_*A*_ is zero in strictly recombining populations and 1 in strictly clonal populations. *I*_*A*_ was calculated using the LIAN 3.5 software [35].

## Results

### Identification of the species of the *Coccidioides* spp. isolates

All the sequences obtained (accession no. JQ919960-JQ919967; JQ919969-JQ919977; JQ919979-JQ919994) were located between positions 902 and 1304 of *Ag2/PRA* gene in the *C. posadasii* genome (accession no. AF013256). The 32 isolates of *Coccidioides* spp. from MX and AR were identified as *C. posadasii* with a nucleotide identity > 99%, trough of the phylogenetic inference analysis (Additional file 3).

### Phenotypic variation

#### Macromorphology

The isolates presented the macromorphology typically described for the *C. posadasii*. The texture of the colonies was generally fuzzy with smooth edges; several colonies had a powdery appearance. For the majority of the isolates, the colour of the front was white with buff components. The reverse side for most of the isolates was buff; however, isolates 073089, HU12 and 083378 exhibited a brown pigmentation (data not shown).

#### Growth rates

The *C. posadasii* isolates grown in YEG-agar medium containing 4 and 6% NaCl had lower growth rates compared with controls (no NaCl) by a statistically significant margin (*p* < 0.01), whereas the isolates grown in medium containing 2% NaCl did not show statistically significant differences in growth rates compared to the controls. No statistically significant differences were observed after conducting analyses by country of origin (Table 1).

**Table 1:** **Growth rates of the *C. posadasii *isolates in different NaCl concentrations**

**Growth rates (cm/day)**				
**Isolate**	**NaCl (0%)**	**NaCl (2%)**	**NaCl (4%)**	**NaCl (6%)**
M0104	3.48	3.55	2.28	1.4
M1204	3.45	3.11	1.63	0.75
M1404	2.83	2.9	1.58	0.66
M1505	2.85	2.8	1.93	1.06
M2305	2.18	2.15	1.6	1.03
M2805	3.01	2.95	2.03	1.23
M3005	2.35	2.46	1.31	0.98
M3905	3.68	3.4	1.8	1.06
M5406	2.91	3.6	2.5	1.13
M5708	2.7	3.51	2.23	1.31
HU1	3.58	3.78	2.58	1.3
HU2	3.13	3.91	2.1	1.05
HU11	3.63	3.28	2.23	1.43
HU12	3.56	3.65	2.43	1.13
HU18	3.21	3.15	2	1.16
HU19	2.26	2.6	1.63	0.9
HU24	3.45	3.5	2.06	1.26
37.3	3.5	3.8	2.45	1.48
5256	2.88	2.5	2	2.86
MA	3.45	3.4	2.1	1.01
SiFe	3.11	3.36	2.2	1.18
972579	3.05	3.35	2.11	1.26
073089	3.68	3.48	2.3	1.26
073094	3.53	3.1	2.16	1.1
073129	3.58	3.6	2.13	1.11
073130	2.88	2.86	1.46	0.95
073131	3.73	3.48	2.4	1.36
083376	2.65	2.91	1.61	0.9
083377	3.53	3.25	2.26	2.5
083380	3.16	3.46	1.93	1.28
083382	2.43	3.08	1.78	1.11
083381	3.05	2.96	2.73	1.05

#### Conidial size

The arthroconidial sizes for the *C. posadasii* isolates ranged from 2.54 to 3.37 μm in width and 4.35 to 8.80 μm in length. Isolate HU12 had the largest arthroconidial size, with arthroconidia measuring 2.56 × 8.80 μm; however, statistically significant differences in arthroconidial size between HU12 and the other samples tested were not observed (*p* < 0.05).

### Genotypic variability and population structure

#### AFLP data analyses

Six primer combinations yielded 170 amplified AFLP markers. The levels of genetic variation of *C. posadasii* from the MX and AR populations are shown in Table 2. In general, the MX and AR isolates had high genetic variability, as demonstrated by the calculated values for the different genetic diversity estimators (the effective number of alleles, the average heterogeneity and the Shannon Index (I), which is insensitive to the number of isolates analysed). The AMOVA showed that 95.21% of the molecular variance was distributed within the *C. posadasii* populations from MX and AR. Our results showed a small genetic differentiation between *C. posadasii* isolates of both countries (*Fst* = 0.048, *p* = 0.0001).

**Table 2:** **Polymorphism, effective number of alleles, genetic diversity and expected heterozygosity of *C. posadasii *isolates from MX and AR**

**Populations**	**P(%)**	***n***_**e**_	***I***	***h***
*C. posadasii* (MX)	91.76	1.5970 ± 0.0603	0.5011 ± 0.0382	0.3396 ± 0.0289
*C. posadasii* (AR)	71.76	1.4754 ± 0.0694	0.3951 ± 0.0503	0.2687 ± 0.0361

P (polymorphism); *n*_*e*_ (effective number of alleles); *I* (genetic diversity, Shannon´s index); *h* (heterozygosity).

MX (Mexico); AR (Argentina).

**Figure 1:**
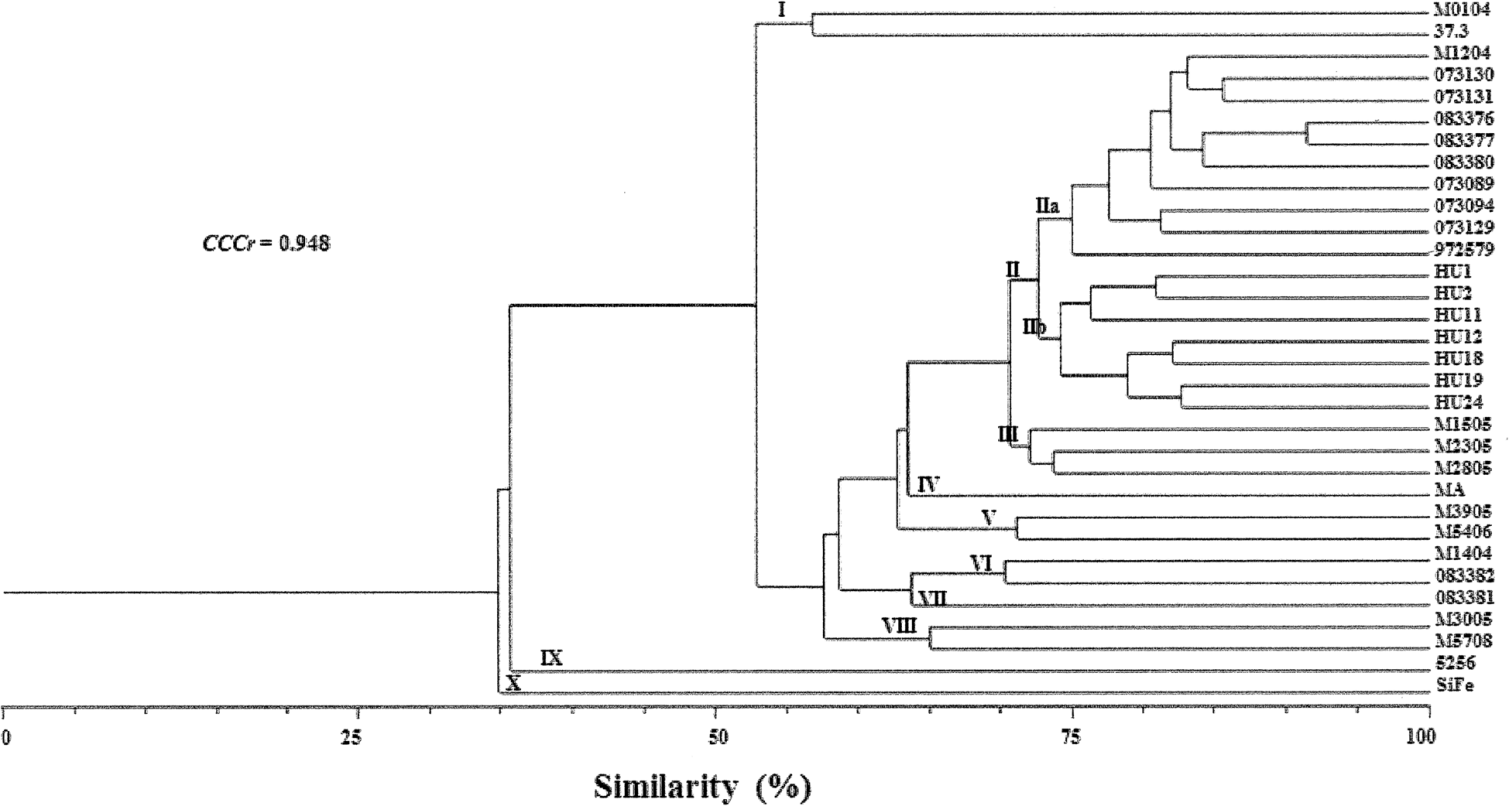
**Dendrogram generated of the *C. posadasii *isolates from MX and AR constructed using the paired genetic distances algorithm of UPGMA.**

Furthermore, the dendrogram for the *C. posadasii* isolates yielded 10 clusters (Figure 1). The first cluster included two isolates from MX, with 56% similarity. Group II included 17 isolates from MX and AR with 70% similarity and showed two subgroups. Subgroup IIa included 9 isolates from AR and one clinical isolate from MX (isolate M1204) with 74% similarity, and subgroup IIb included 7 isolates from MX, with 73% similarity. Group III contained three isolates from MX with 73% intragroup similarity. Group IV consisted of one isolate from MX with 62% intragroup similarity. Group V consisted of two isolates from MX with 71% similarity. Group VI consisted of two isolates, one from MX and another from AR, with 70% similarity. Group VII included only one isolate (isolate 083381) from AR, with 63% similarity to group VI. Group VIII included two isolates from MX, with 65% similarity. Group IX consisted of only one isolate from MX, with 36% similarity with the above. Finally, group X included only one isolate from MX, with 35% similarity. The cophenetic correlation coefficient (CCC*r* = 0.948, *p* = 0.0004) suggested that the dendrogram accurately represented the original genetic similarities among the isolates.

The network of haplotypes demonstrated a large number of crosslinks among *C. posadasii* isolates and showed a small differentiation between the isolates from MX and AR.

**Figure 2:**
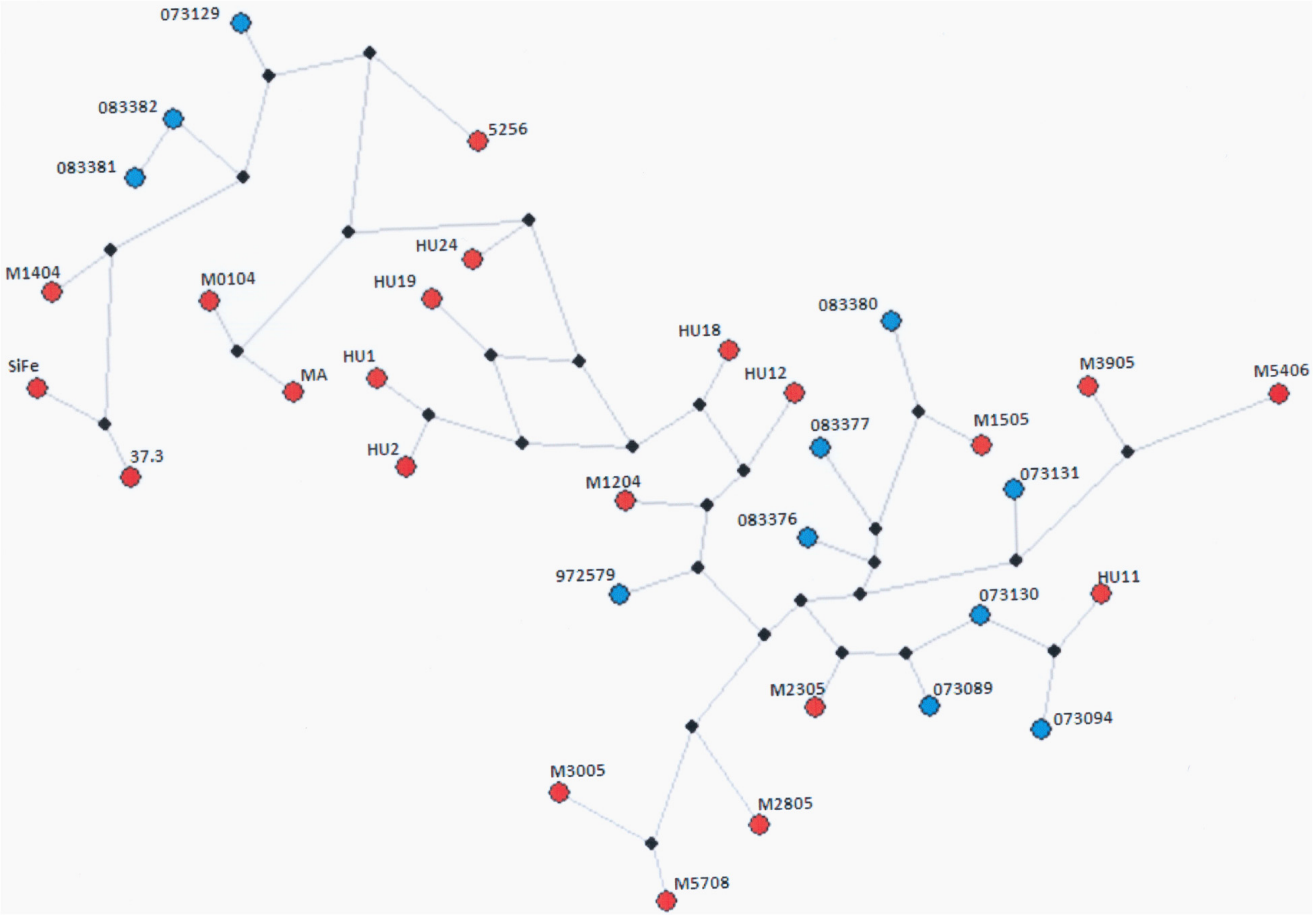
**Analyses of haplotype networks for *C. posadasii *isolates using the median-joining method.** The red circles correspond to isolates from MX, the blue circles correspond to isolates from AR.

Additionally, the number of mutations between each node (1–30) in the haplotype network demonstrated broad genetic variability (Figure 2).

Finally, the *I*_*A*_ (0.0287) calculated for the isolates in this study suggested that these isolates have a recombinant reproductive system.

## Discussion

The spread of coccidioidomycosis over recent years in endemic areas of MX and AR is sufficient to warrant attention given that there are so few studies of the disease. Therefore, it is important to be well informed about the different aspects of *Coccidioides* spp*.* to carefully manage the disease. In this study, we estimated the genetic variability among *C. posadasii* isolates from MX and AR. Our results indicate that the partial *Ag2/PRA Coccidioides* spp. sequences obtained by Bialek *et al*. [21] method are useful for identify *C. posadasii* from different geographical origins. This fragment can also be used to identify *C. immitis* because this species presents in this region a deletion of 12 bases [36]. Thus, this fragment is not suitable to diversity or genetic structure studies, because it presents scarce variation. On the other hand, the results of phenotypic characterisation parameters (growth rates using different NaCl concentrations and arthroconidial size) showed no differences among the *C. posadasii* isolates.

The genetic diversity of the *C. posadasii* isolates from MX and AR showed high genetic variability using polymorphic AFLP. However, AMOVA indicated that this variation was not geographically structured. The results from this study suggested high rates of gene flow between isolates in MX and AR, which explains the scarce differentiation found among them. A probable explanation about these findings may be the constant flow of genes between these populations favoring the air-borne dissemination of the fungus since the hyphae that constitute the saprobe or infectious stage fragment even with the lightest air currents, freeing the arthroconidia and easily travelling large distances in the wind [37,38]. The viability of the conidia in the environment is favored by the tolerability of the fungi to high temperatures (50°C) and their resistance to UV light (due to their melanine content) benefiting survival and longevity. Another possible explanation maybe high gene flow is a consequence of the constant movement of people across the continent or due to the migration of mammals [39], among these specific bat species which are long-distance migratory. This hypothesis is supported by the recent findings by Cordeiro *et al*. [40] who demonstrated the infection by *Coccidioides* spp. in these mammals.

The no-association among the *C. posadasii* isolates from MX and AR with their geographical origin, was supported by the dendrogram and haplotype network, confirm the scarce genetic differentiation observed between them. These findings partially concur with Fisher *et al*. [39], who found low variability among *Coccidioides* spp. isolates and little genetic differentiation among isolates from South America and the US. Notwithstanding, the technique used in this study was the AFLP. This is a useful tool for establishing the changes in the genome of the fungi isolates allowing for simultaneously analysing many loci and detecting a greater number of polymorphic DNA markers than any other method based on PCR. However, a limiting factor in this study was the impossibility of comparing results with other studies on the structure of *Coccidioides* spp. populations due to the use of different methodologies. It is recommended that future studies use markers that have been validated and employed by other authors in studies of genetic variability of *Coccidioides* spp. in order to compare the results obtained with isolates from different geographic regions.

On the other hand, the high genetic variability found among the isolates studied maybe explained by a recombinant sexual reproductive system (*I*_*A*_*=* 0.0287), as was suggested by other authors [1,13-17,41,42]. This mode of reproduction was also supported by the presence of the potentially functional MAT idiomorph loci, MAT1-2 (HMG) and MAT1–1 (alpha-box) [41,42]. Thus, even though this species’ sexual phase remains undescribed, studies indicate that, based on high variability, these fungi recombine, gaining advantages such as adaption to new environments; thus, virulent or resistant strains could emerge, which may also explain the numerous recent epidemic outbreaks.

The observed high variability may also be explained by the possibility that the different genotypes could adapt to other environments under inappropriate developmental conditions, which is true for the isolate “MA”, found in a patient originally from the state of Campeche, located in the Southeast region of MX, who claimed to have never left the state. This region has climatic conditions different from the preferred fungal growth conditions. Similarly, adaptation to new environments facilitates the appearance of hypervirulent strains, as suggested by Fisher *et al*. [1] and Jewell *et al*. [18].

It is important to understand variability among these fungi, which has also been investigated in recent publications, to determine genotype distributions among populations, monitor outbreaks, assess variations in virulence and predict disease progression [18,43,44]. Several lines of research that pertain to this issue are in progress that will further our understanding of the underlying biology of these pathogens and their interactions with other living species.

## Conclusions

Phenotypic characterisation indicated no differences among the *C. posadasii* isolates studied. The different estimators of genetic variability employed indicated that the isolates from MX and AR had high genetic variability. The AMOVA showed a small genetic differentiation among the *C. posadasii* populations analysed from MX and AR; thus, these populations were genetically similar. Furthermore, the *I*_*A*_ calculated for the isolates suggested that the *C. posadasii* isolates had recombinant reproductive systems, which would contribute to the high variability found among the isolates.

## Competing interests

The authors declare that they have no competing interests.

## Authors’ contributions

MRRM, EDE were involved in the study design, analised and interpreted the results and drafted the manuscript. EDE and MGFDL performed the experiments. GZ conducted data analysis and participated in drafting the manuscript and provided a critical review of the manuscript. CC participated in the study design and provided a critical review of the manuscript. RCO performed microscopic fungal identification. MRRM conceptualized and coordinated the project. All authors read and approved the final manuscript.

## Pre-publication history

The pre-publication history for this paper can be accessed here:

http://www.biomedcentral.com/1471-2334/13/411/prepub

## Supplementary Material

Additional file 1 **Source and geographic origin of *Coccidioides posadasii *isolates.** Click here for file

Additional file 2 **Binary data matrix.** Each AFLP band was treated as a separate character and scored 1 (present) or 0 (absent). Click here for file

Additional file 3 **The 32 isolates of *Coccidioides *spp. from MX and AR were identified as *C. posadasii *trough of the phylogenetic inference analysis.** Click here for file
