# Supplementary material for: AFLP analysis reveals high genetic diversity but low population structure in Coccidioides posadasii isolates from Mexico and Argentina
Source: BMC Infect Dis. 2013 Sep 3;13:411. doi: 10.1186/1471-2334-13-411 (PMC3766708; doi:10.1186/1471-2334-13-411)
Supplement: Additional file 1 — Source and geographic origin of Coccidioides posadasii isolates. [file 1471-2334-13-411-S1.pdf]

Additional file 1. Source and geographic origin of *Coccidioides posadasii* isolates.

Isolates	Source	Origin
M0104	Catheter (Peritoneal)	Distrito Federal, MX
M1204	Sputum	Durango, MX
M1404	Cerebrospinal fluid	Torreón, MX
M1505	Cerebrospinal fluid	Torreón, MX
M2305	Pleural secretion	Torreón, MX
M2805	Bronchoalveolar lavage	Torreón, MX
M3005	Sputum	Torreón, MX
M3905	ND	Baja California, MX
M5406	ND	Tijuana, MX
M5708	ND	San Luis Potosí, MX
HU1	ND	Monterrey, MX
HU2	ND	Monterrey, MX
HU11	Bronchoalveolar lavage	Monterrey, MX
HU12	Bronchoalveolar lavage	Monterrey, MX
HU18	Articular secretion	Monterrey, MX
HU19	Bronchial secretion	Monterrey, MX
HU24	Sputum	Monterrey, MX
37.3	ND	MX
5256	ND	MX
MA	Bronchoalveolar lavage	Campeche, MX
SiFe	ND	MX
972579	Node biopsy	Catamarca, AR

073089	Bronchoalveolar lavage	Catamarca, AR
073094	Cerebrospinal fluid	Mendoza, AR
073129	Skin biopsy	Salta, AR
073130	Blood culture	Catamarca, AR
073131	Lung biopsy	La Rioja, AR
083376	Bronchoalveolar lavage	Catamarca, AR
083377	Skin biopsy	Catamarca, AR
083380	Leg abscess	Catamarca, AR
083382	Node biopsy	Santiago del Estero, AR
083381	Sputum	Catamarca, AR

ND-undeterminated; MX-Mexico; AR-Argentina
