# Supplementary material for: AFLP analysis reveals high genetic diversity but low population structure in Coccidioides posadasii isolates from Mexico and Argentina
Source: BMC Infect Dis. 2013 Sep 3;13:411. doi: 10.1186/1471-2334-13-411 (PMC3766708; doi:10.1186/1471-2334-13-411)
Supplement: Additional file 2 — Binary data matrix. Each AFLP band was treated as a separate character and scored 1 (present) or 0 (absent). [file 1471-2334-13-411-S2.pdf]

Additional file 2. Binary data matrix. Each AFLP band was treated as a separate character and scored 1 (present) or 0 (absent).

	A	B	C	D	E	F	G	H	I	J	K	L	M	N	O	P	Q	R	S	T	U	V	W	X	Y	Z	AA	AB	AC	AD	AE	AF	AG
1	1	170	32																														
2		M010	M120	M140	M1505	M2305	M280	M3005	M390	M540	M5708	HU1	HU2	HU11	HU12	HU18	HU19	HU24	MA	37.	5256	SiF	####	07308	07309	073129	07313	07313	08337	08337	08338	08338	083381
3		999	1	1	0	1	1	0	1	0	1	1	1	1	1	1	1	1	1	1	1	1	1	1	1	1	1	1	1	1	1	1	1
4		999	0	0	0	0	0	0	0	0	0	1	1	0	1	1	1	0	0	1	0	0	0	0	1	0	0	0	0	0	0	1	1
5		999	1	0	0	1	1	0	1	0	0	1	1	1	1	1	1	0	0	0	0	0	1	1	1	1	1	1	1	1	0	0	0
6		999	1	1	0	1	1	0	1	1	0	1	1	1	1	1	1	1	0	0	0	0	1	1	1	1	1	1	1	1	0	0	1
7		999	1	1	1	1	1	1	0	0	0	1	1	1	1	0	0	0	0	0	0	0	1	1	1	1	1	1	1	1	0	1	1
8		999	0	1	0	0	0	0	1	1	0	0	0	0	1	1	1	1	0	0	0	1	0	0	0	0	0	0	0	0	0	1	1
9		999	1	1	1	1	1	0	1	1	1	1	1	1	1	1	1	1	1	1	1	1	1	1	1	1	1	1	1	1	1	1	1
10		999	1	1	0	1	1	0	1	1	0	1	1	1	1	1	1	1	0	1	0	1	1	1	1	1	1	1	1	1	1	1	1
11		999	1	1	0	1	1	1	1	1	0	1	1	1	1	1	1	1	0	1	0	1	1	1	1	1	1	1	1	1	1	1	1
12		999	0	1	0	0	1	0	1	1	0	1	1	1	1	1	1	1	0	0	0	0	1	1	1	1	1	1	1	1	0	1	1
13		999	1	0	0	1	0	0	0	1	0	1	1	1	1	0	0	0	0	1	0	0	0	0	1	0	1	1	1	1	1	0	0
14		999	0	0	0	0	0	0	1	1	1	0	0	0	1	1	1	1	1	0	0	1	1	0	1	0	1	1	1	1	0	0	0
15		999	1	1	0	0	0	0	1	1	0	0	0	0	0	0	0	0	0	0	0	0	1	1	1	1	1	1	1	1	1	1	0
16		999	0	0	0	0	0	0	0	1	0	1	1	1	1	0	1	0	0	0	0	1	0	0	0	0	0	0	0	0	0	1	1
17		999	1	1	0	1	1	0	1	1	0	1	1	0	0	1	1	1	0	0	0	0	0	1	1	0	1	1	1	0	1	0	0
18		999	0	1	0	0	0	0	1	1	0	0	1	0	1	1	1	1	0	0	0	0	1	1	1	0	1	1	1	1	0	1	0
19		999	1	0	1	1	1	1	1	1	0	1	1	1	1	1	0	0	0	0	0	0	1	0	1	0	1	1	1	1	0	0	0
20		999	1	1	0	0	1	1	0	1	1	1	1	1	1	0	1	1	1	1	0	0	1	1	1	1	1	1	1	1	0	1	1
21		999	1	0	1	1	0	0	1	1	0	1	1	1	1	1	1	1	0	0	0	0	1	1	1	0	1	1	1	1	0	0	0
22		999	1	1	1	1	1	1	1	1	1	1	1	1	1	1	1	1	1	1	0	0	1	1	1	1	1	1	1	1	1	1	1
23		999	1	0	0	1	0	0	1	1	1	1	1	0	0	1	1	1	1	1	0	1	0	0	1	0	1	1	1	0	0	1	1
24		1	1	1	1	1	1	1	1	1	1	1	1	1	1	1	1	1	1	1	999	0	1	1	1	999	1	1	1	1	1	1	1
25		0	0	0	0	0	0	0	0	0	0	0	0	0	0	0	1	0	0	0	999	0	0	0	0	999	0	0	0	0	0	0	1
26		1	1	1	1	1	1	1	1	1	1	1	1	1	1	1	1	1	1	1	999	0	1	1	1	999	1	1	1	1	1	1	1
27		0	1	0	1	0	1	1	1	1	1	1	1	0	1	1	1	1	0	0	999	0	1	0	0	999	0	0	1	1	1	0	1
28		1	0	0	0	0	0	0	1	1	0	1	1	0	1	0	1	1	0	0	999	0	0	0	0	999	0	0	0	0	0	1	1
29		1	0	0	0	0	0	0	1	1	0	0	0	0	0	0	0	0	0	0	999	0	0	0	0	999	0	0	0	0	0	0	1
30		0	1	0	1	1	1	1	1	1	1	1	1	1	1	1	1	1	1	0	999	0	1	1	1	999	1	1	1	1	1	1	1
31		0	0	0	1	0	0	1	0	0	1	1	1	0	1	1	0	0	0	0	999	0	0	0	0	999	0	0	0	0	0	0	1
32		0	1	0	1	1	1	1	1	1	0	1	1	0	1	1	1	1	0	0	999	0	0	0	0	999	1	1	1	1	1	0	1
33		1	1	1	1	0	1	0	1	0	1	1	1	1	1	1	1	1	0	0	999	0	1	0	1	999	0	1	1	1	1	0	1
34		0	0	0	1	1	1	1	0	1	0	0	0	0	0	0	0	0	1	0	999	0	0	0	0	999	0	0	0	0	0	0	1
35		1	1	0	1	1	1	1	1	1	1	1	1	1	1	1	1	1	1	0	999	0	1	1	1	999	1	1	1	1	1	1	1
36		0	0	0	0	0	0	0	0	1	0	1	1	0	0	0	0	0	0	0	999	0	1	0	0	999	0	0	0	0	0	0	1
37		0	1	0	1	0	1	1	1	0	1	0	1	1	1	1	1	1	1	0	999	0	1	1	0	999	1	1	1	1	1	0	1
38		0	1	0	1	0	0	1	0	1	1	0	1	0	1	1	0	1	0	0	999	0	0	0	0	999	0	0	1	1	1	0	1
39		0	1	0	1	1	1	1	1	0	0	0	1	0	1	0	1	1	0	0	999	0	0	0	0	999	0	1	1	1	1	0	1
40		1	1	1	1	1	1	1	1	1	1	1	1	1	1	1	1	1	1	1	999	0	1	1	1	999	1	1	1	1	1	1	1
41		0	1	0	1	1	1	1	0	0	1	1	1	0	1	0	1	1	0	0	999	0	0	1	0	999	1	1	1	1	1	0	1
42		0	0	0	1	0	0	0	0	0	0	0	0	0	1	1	1	0	0	0	999	0	0	0	0	999	0	0	0	0	0	1	1
43		0	0	0	1	1	1	1	0	1	1	1	1	0	0	0	0	1	0	0	999	0	0	1	0	999	1	0	1	1	1	0	0
44		1	1	1	1	1	1	1	1	1	1	1	1	1	1	1	1	1	1	1	999	1	1	1	1	999	1	1	1	1	1	1	1
45		0	1	0	1	1	1	1	1	0	1	1	1	1	1	1	1	1	1	0	999	0	1	1	1	999	1	1	1	1	1	0	1
46		0	1	1	1	1	1	1	1	1	1	1	1	1	1	1	1	1	1	0	999	0	1	1	1	999	1	1	1	1	1	1	1
47		0	0	0	0	0	0	0	0	1	0	0	0	0	0	0	0	0	0	0	999	0	0	0	0	999	0	0	0	0	0	0	1
48		1	1	1	1	1	1	1	1	0	1	1	1	1	1	1	1	1	1	1	999	0	1	1	1	999	1	1	1	1	1	1	1
49		0	0	0	0	0	0	1	0	1	0	0	0	0	0	0	0	0	0	0	999	0	0	0	0	999	0	0	0	0	0	0	1
50		0	0	0	0	0	1	1	0	1	0	1	1	0	1	1	1	1	0	0	999	0	0	0	0	999	0	0	0	0	0	0	0
51		1	1	0	1	0	1	1	1	1	1	1	1	1	1	1	1	1	0	1	999	0	1	1	1	999	1	1	0	1	1	1	1
52		1	1	0	1	0	1	0	1	1	1	0	0	0	1	1	0	0	0	0	999	0	0	1	1	999	1	1	1	1	1	0	1
53		0	0	0	0	0	0	0	0	1	0	0	0	0	0	0	0	0	0	0	999	0	0	0	0	999	0	0	0	0	0	0	1
54		1	1	1	1	1	0	1	1	1	1	1	1	1	1	1	1	1	1	0	999	0	1	1	1	999	1	1	1	1	1	1	1
55		0	0	0	1	0	0	0	1	1	0	0	0	0	0	0	0	0	0	0	999	0	0	0	0	999	0	0	0	0	0	0	1
56		0	0	0	1	0	1	1	1	1	1	1	1	1	1	1	1	1	1	0	999	0	0	1	0	999	0	0	0	1	1	0	1
57		1	1	0	1	1	1	1	1	1	1	1	1	1	1	1	1	1	1	0	999	0	1	1	0	999	1	1	1	1	1	0	1
58		1	0	0	1	1	1	1	0	1	1	1	0	1	1	1	1	1	0	0	999	0	1	1	0	999	1	1	1	1	1	0	1
59		1	0	0	0	1	0	1	0	1	1	1	0	1	0	1	1	1	0	0	999	0	1	0	0	999	0	0	0	0	0	1	1
60		1	1	1	1	1	1	1	0	0	1	0	1	1	1	1	1	1	1	1	999	0	1	1	1	999	1	1	1	1	1	1	1
61		0	0	0	1	1	1	0	1	1	1	1	1	1	1	1	1	1	1	0	999	0	1	1	1	999	1	1	1	1	0	0	1
62		1	0	0	0	0	0	0	0	1	0	0	0	0	0	0	0	0	0	0	999	0	1	0	0	999	0	0	0	0	0	1	1
63		1	1	1	1	1	1	1	1	1	1	1	1	1	1	1	1	1	1	1	1	1	1	1	1	1	1	1	1	1	1	999	1
64		1	1	1	1	1	1	1	1	1	1	1	1	1	1	1	1	1	1	1	0	0	1	1	1	1	1	1	1	1	1	999	1
65		0	0	0	0	0	0	0	1	1	0	0	0	0	0	0	0	0	0	1	0	0	0	0	0	0	0	0	0	0	0	999	0
66		0	0	1	0	0	0	0	1	1	0	0	0	0	0	0	0	0	0	1	0	1	0	0	0	0	0	0	0	0	0	999	0
67		0	0	0	0	0	0	0	1	1	0	0	0	0	0	0	0	0	0	0	0	0	0	0	0	0	0	0	0	0	0	999	0
68		0	1	1	1	1	1	1	1	1	1	1	1	1	1	1	1	1	1	1	1	1	1	1	1	1	1	1	1	1	1	999	1
69		0	0	1	0	0	0	0	1	1	0	0	0	0	0	0	0	0	0	1	1	0	0	0	0	0	0	0	0	0	0	999	1
70		0	1	0	1	1	1	0	1	1	0	0	1	1	1	0	0	0	0	0	0	1	0	0	0	1	1	1	1	1	1	999	1
104		1	1	1	1	1	1	1	1	1	1	1	1	1	1	1	0	1	1	1	1	0	0	1	1	1	1	1	1	1	1	1	1
103		1	1	1	1	1	1	1	1	1	1	1	1	1	1	1	1	1	1	1	1	1	0	1	1	1	1	1	1	1	1	1	1
102		1	0	0	0	0	0	0	1	1	0	0	0	0	0	0	0	0	0	0	1	0	0	0	0	1	0	1	1	1	0	1	0
101		1	0	1	0	0	0	0	1	1	0	0	0	0	0	0	0	0	1	1	1	0	0	1	1	1	1	1	0	0	1	1	1
100		1	1	0	1	1	1	0	1	1	0	1	0	1	1	1	1	1	0	1	1	0	0	1	1	1	1	1	1	1	1	1	0
99		1	0	1	0	0	0	0	1	1	1	0	0	1	1	1	1	1	1	0	1	1	0	1	0	1	1	1	1	1	1	1	1
98		1	1	0	1	1	1	1	1	1	0	1	0	0	1	0	0	0	1	0	1	0	0	1	1	1	1	1	1	1	1	1	0
97		1	1	0	1	0	0	0	1	1	0	1	0	0	1	1	1	1	1	0	1	0	1	1	1	1	1	1	1	1	1	1	1
96		1	1	1	1	1	1	1	1	1	1	1	1	1	1	1	1	1	1	1	1	1	1	1	1	1	1	1	1	1	1	1	1
95		1	1	0	0	0	1	0	0	0	0	0	0	0	0	0	0	0	0	0	0	0	0	1	1	0	0	0	1	0	1	0	0
94		1	1	1	1	1	1	1	1	1	1	1	1	1	1	1	1	1	1	1	1	0	1	1	1	1	1	1	1	1	1	1	1
93		1	1	1	1	1	1	1	1	1	1	1	0	1	1	1	1	1	0	1	1	1	1	1	1	1	1	1	1	1	1	1	1
92		1	0	0	0	0	0	0	1	1	0	0	0	0	0	0	0	0	0	1	0	0	0	0	0	0	0	0	0	0	0	0	0
91		1	1	1	1	0	0	1	1	1	0	1	1	1	1	1	1	0	0	1	1	0	1	1	1	1	1	1	1	1	1	1	1
90		1	1	0	1	0	0	1	1	1	0	1	1	1	1	1	1	0	0	0	0	0	1	1	1	1	1	1	1	1	1	0	0
89		1	1	1	1	1	1	1	1	1	1	1	1	1	1	1	1	1	1	1	1	1	1	1	1	1	1	1	1	1	1	1	1
88		1	1	1	1	1	1	1	1	1	1	1	1	1	1	1	1	1	1	1	1	1	1	1	1	1	1	1	1	1	1	1	1
87		1	1	1	1	1	1	1	1	1	1	1	1	1	1	1	1	1	1	0	0	0	1	1	1	1	1	1	1	1	1	999	1
86		1	1	0	1	1	1	0	1	1	0	0	1	0	0	0	0	0	0	0	0	0	1	1	0	1	0	1	1	1	1	999	0
85		0	1	1	1	1	1	0	0	0	0	0	1	0	1	0	0	0	0	1	0	0	1	1	0	1	0	1	1	1	1	999	0
84		1	1	1	1	0	1	1	0	0	0	1	1	0	1	1	1	1	1	1	0	1	1	1	1	1	1	1	1	1	1	999	1
83		0	0	0	1	1	0	0	1	0	0	0	0	0	1	1	1	0	0	1	0	0	1	1	0	1	0	0	1	1	1	999	0
82		1	1	1	1	1	1	0	1	1	0	0	0	0	1	1	1	1	1	1	0	1	1	1	0	1	1	1	1	1	1	999	1
81		1	1	1	1	0	1	1	0	0	1	1	0	0	1	1	1	1	1	1	0	1	1	1	1	1	1	1	1	1	1	999	1
80		1	1	0	1	1	1	1	1	1	1	1	1	1	1	1	1	1	1	1	0	0	1	1	1	1	1	1	1	1	1	999	0
79		0	1	0	1	1	1	1	1	1	0	1	1	1	1	1	1	1	1	0	0	0	1	1	1	1	1	1	1	1	1	999	0
78		1	1	1	1	1	1	1	1	1	0	1	1	1	1	1	1	1	1	1	0	0	1	1	1	1	1	1	1	1	1	999	1
77		1	1	1	1	1	1	1	0	0	1	1	1	1	1	1	1	1	1	1	0	1	1	1	1	1	1	1	1	1	1	999	0
76		1	1	1	1	1	1	1	1	1	1	1	1	1	1	1	1	1	1	1	0	0	1	1	1	1	1	1	1	1	1	999	1
75		0	0	1	1	1	0	0	0	0	0	0	0	0	0	0	0	0	0	1	0	1	0	0	0	0	0	0	0	1	1	999	1
74		0	0	1	1	1	0	0	0	0	0	0	0	0	0	0	0	0	0	0	0	1	0	0	0	0	0	0	0	1	1	999	1
73		1	1	1	1	1	1	0	1	1	0	0	1	0	1	0	0	1	0	1	0	0	1	0	1	1	0	1	1	1	1	999	1
72		1	1	0	1	1	1	1	1	1	1	1	1	1	1	1	1	1	1	0	0	1	1	1	1	1	1	1	1	1	1	999	0
71		0	0	1	0	0	0	0	0	0	0	0	0	0	0	0	0	0	0	1	1	0	0	0	0	0	0	0	1	1	0	999	1

139	1	1	1	1	1	1	1	1	1	1	1	1	1	1	1	1	1	1	1	999	1	1	1	1	1	1	1	1	1	1	1	1
138	0	1	1	0	1	1	0	0	1	1	1	1	1	1	1	1	1	1	1	999	0	1	1	1	1	1	1	1	1	1	1	1
137	1	0	0	0	0	0	0	1	0	0	0	0	0	0	0	0	0	0	0	999	1	0	0	0	0	0	0	0	0	0	0	0
136	0	1	1	1	1	1	0	1	1	0	1	1	1	1	1	1	1	1	1	999	1	1	1	1	1	1	1	1	1	1	1	1
135	1	1	1	1	1	1	0	1	1	0	1	1	1	1	1	1	1	1	1	999	1	1	1	1	1	1	1	1	1	1	1	0
134	0	0	0	0	0	0	0	0	0	0	1	0	1	0	0	1	1	0	0	999	0	0	0	1	1	0	0	0	0	0	0	0
133	1	1	1	1	1	1	1	1	1	1	1	1	1	1	1	1	1	1	1	999	1	1	1	1	1	1	1	1	1	1	1	1
132	0	0	1	0	1	0	0	0	0	0	1	1	1	0	0	1	1	0	0	999	0	0	0	1	1	1	0	1	0	1	1	0
131	0	1	0	0	0	0	0	0	1	0	1	1	1	0	0	0	0	0	0	999	0	0	0	0	0	0	0	1	0	1	0	0
130	0	0	0	0	0	0	0	0	1	0	0	0	0	0	0	0	0	0	0	999	0	0	0	0	0	0	0	0	0	0	0	0
129	0	1	0	1	1	1	1	1	1	1	1	1	1	1	1	1	1	1	0	999	0	1	1	1	1	1	1	1	1	1	1	1
128	1	1	1	1	1	1	1	1	1	1	1	1	1	1	1	1	1	1	1	999	1	1	1	1	1	1	1	1	1	1	1	1
127	0	0	1	0	0	0	0	0	0	0	0	1	0	0	0	0	0	0	0	999	0	0	0	0	0	0	0	1	1	0	0	0
126	0	1	0	1	0	0	0	0	1	1	0	0	1	1	1	1	1	1	0	999	1	1	1	1	1	1	1	1	1	1	0	0
125	0	0	0	0	0	0	0	0	1	0	0	0	0	0	0	0	0	0	0	999	0	0	0	0	0	0	0	0	0	0	0	0
124	1	1	1	1	1	1	1	1	1	1	1	1	1	1	1	1	1	1	1	999	1	1	1	1	1	1	1	1	1	1	1	1
123	0	1	0	1	0	0	0	0	1	0	0	1	1	0	0	0	1	1	0	999	0	1	1	0	1	1	1	1	1	1	0	0
122	0	0	1	0	0	0	0	0	1	0	1	0	0	0	0	1	1	1	0	999	0	0	0	0	0	0	0	0	0	0	1	1
121	0	1	0	0	1	0	0	0	0	0	1	1	1	1	1	0	1	0	0	999	1	0	0	1	1	1	1	1	1	1	0	1
120	0	0	1	0	1	0	0	0	0	0	1	1	0	0	0	0	0	0	0	999	0	0	0	0	0	0	0	0	0	0	1	1
119	1	0	1	1	1	1	1	1	1	1	1	1	1	0	1	1	1	1	0	999	0	1	1	1	1	1	1	1	1	1	1	1
118	0	0	1	0	0	0	0	0	0	0	0	0	0	0	0	0	0	0	0	999	0	0	0	1	1	0	0	0	0	0	1	1
117	0	1	1	1	1	1	1	0	0	1	1	1	1	1	1	1	1	0	0	999	0	1	1	1	1	1	1	1	1	1	0	0
116	0	1	0	1	1	1	1	1	1	1	1	1	1	1	1	1	1	0	0	999	1	1	1	1	1	1	1	1	1	1	0	1
115	1	1	1	1	1	1	1	1	1	1	1	1	1	1	1	0	1	0	0	999	0	1	1	1	1	1	0	1	1	1	0	0
114	0	0	1	0	1	0	0	0	0	0	1	1	1	1	1	1	1	0	0	999	0	0	0	1	1	1	0	1	0	1	1	0
113	1	1	1	1	1	0	0	0	1	1	1	1	1	1	1	1	1	1	0	999	0	1	0	1	1	1	0	1	1	1	1	1
112	1	1	1	1	1	0	0	0	1	0	1	1	1	1	1	1	1	1	1	999	0	1	1	1	1	1	1	1	1	1	1	1
111	0	1	0	0	1	1	1	1	1	1	1	1	1	1	1	1	1	1	0	999	1	1	1	1	1	1	1	1	1	1	1	1
110	1	1	1	1	1	1	1	1	1	1	1	1	1	1	1	1	1	1	1	999	1	1	1	1	1	1	1	1	1	1	1	1
109	1	1	1	1	1	0	0	1	1	1	1	1	1	1	1	1	1	1	0	999	0	1	1	1	1	1	1	1	1	1	1	1
108	0	1	1	1	1	0	0	1	1	0	1	1	1	1	1	1	1	1	0	999	0	0	1	1	1	1	1	1	0	1	1	0
107	1	1	1	1	1	1	1	1	1	1	1	1	1	1	1	1	1	1	1	999	1	1	1	1	1	1	1	1	1	1	1	1
106	1	0	1	0	0	0	0	1	0	1	0	0	0	0	0	1	1	1	1	1	0	0	0	0	0	0	1	1	1	1	1	1
105	1	1	1	1	0	1	1	1	1	1	0	0	1	1	0	0	1	1	1	1	0	0	1	1	1	1	1	1	1	1	1	1
172	0	1	1	1	1	0	0	1	1	1	0	1	1	0	1	0	0	0	0	999	1	0	0	1	0	0	1	0	0	0	0	1
171	0	0	0	0	0	0	0	0	0	0	0	0	0	0	0	0	0	0	0	999	0	0	0	0	0	0	0	0	0	0	1	0
170	1	1	1	1	1	1	1	1	1	1	1	1	1	1	1	1	1	1	1	999	1	1	1	1	1	1	1	1	1	1	1	1
169	1	1	1	1	1	1	1	1	1	1	1	1	1	1	1	1	1	1	1	999	1	1	1	1	1	1	1	1	1	1	1	1
168	1	1	1	1	1	1	1	1	1	1	1	1	1	1	1	1	1	1	1	999	1	1	1	1	1	1	1	1	1	1	1	0
167	1	1	1	1	1	1	1	1	1	0	1	1	1	1	1	1	1	1	1	999	1	1	1	1	1	1	1	1	1	1	1	1
166	1	1	1	1	1	1	1	1	1	1	1	1	1	1	1	1	1	1	1	999	0	1	1	1	1	1	1	1	1	1	1	1
165	1	1	1	1	1	1	1	1	1	1	1	1	1	1	1	1	1	1	1	999	1	1	1	1	1	1	1	1	1	1	1	1
164	0	0	1	0	0	0	0	0	0	0	0	0	1	1	0	0	0	0	0	999	0	0	0	0	0	0	0	0	0	0	1	1
163	1	1	1	1	1	1	1	1	1	1	1	1	1	1	1	1	1	1	1	999	1	1	1	1	1	1	1	1	1	1	1	1
162	1	1	1	1	1	1	1	1	1	0	1	1	1	1	0	1	1	0	1	999	0	1	1	1	1	1	1	1	1	1	1	1
161	0	0	0	0	0	0	0	0	1	0	0	0	0	0	0	0	0	0	0	999	0	0	0	0	0	0	0	0	0	0	0	0
160	0	1	1	0	1	1	0	0	0	0	1	1	1	0	0	0	1	1	1	999	0	1	0	1	1	1	1	1	1	1	1	1
159	1	1	1	0	1	1	0	1	1	1	1	1	1	1	1	1	1	1	1	999	0	1	1	1	1	1	1	1	1	1	1	1
158	0	0	0	0	0	0	0	0	0	0	0	1	1	0	0	0	1	0	1	999	0	0	0	0	0	0	0	0	0	0	1	1
157	0	1	0	1	1	1	0	1	1	0	1	1	1	1	1	1	1	1	0	999	0	1	1	1	1	1	1	1	1	1	1	1
156	0	1	1	1	1	1	0	1	1	1	1	1	1	1	1	1	1	1	1	999	0	1	1	1	1	1	1	1	1	1	1	1
155	1	1	1	1	1	1	1	1	1	1	1	1	1	1	1	1	1	1	1	999	1	1	1	1	1	1	1	1	1	1	1	1
154	0	0	1	1	0	1	1	1	0	1	0	1	1	0	1	0	0	0	0	999	1	1	1	1	1	1	0	1	1	1	1	1
153	0	1	1	1	1	1	1	1	1	1	1	1	1	1	1	1	1	1	1	999	0	1	1	1	1	1	1	1	1	1	1	1
152	0	0	0	0	0	0	0	0	1	0	0	0	0	0	0	0	0	0	0	999	1	0	0	0	0	0	0	0	0	0	0	0
151	1	1	1	1	1	1	1	1	1	1	1	1	1	1	1	1	1	1	1	999	1	1	1	1	1	1	1	1	1	1	1	1
150	1	1	1	1	1	1	1	1	1	1	1	1	1	1	1	1	1	1	1	999	1	1	1	1	1	1	1	1	1	1	1	1
149	1	1	0	1	1	1	1	1	0	1	1	1	1	1	1	1	1	1	0	999	0	1	1	0	1	1	1		1	1	1	1
148	0	0	0	1	1	1	1	0	0	0	1	1	1	0	1	1	1	1	0	999	0	0	0	1	1	0	1	0	0	0	0	0
147	1	1	1	0	0	0	0	1	1	1	0	0	0	1	0	0	0	1	1	999	1	1	1	0	0	1	0	1	1	1	1	1
146	0	1	1	1	1	1	1	0	1	1	1	1	1	1	1	1	1	1	1	999	0	1	0	1	1	1	1	0	0	0	1	1
145	0	0	1	0	0	0	0	1	1	0	1	1	0	0	0	0	1	1	0	999	1	0	0	1	1	0	1	0	0	0	1	1
144	1	1	1	1	1	1	1	1	1	0	1	1	1	1	1	0	1	1	1	999	0	1	1	1	0	1	0	1	1	1	1	1
143	0	1	1	1	1	1	0	1	0	0	1	1	1	1	1	1	1	1	0	999	1	1	1	1	1	1	1	1	1	1	0	0
142	1	1	0	1	1	1	0	0	1	0	1	1	1	1	1	1	1	1	1	999	0	0	0	1	1	1	1	0	0	1	0	0
141	0	1	0	1	0	1	1	1	1	1	0	1	0	1	1	0	1	1	0	999	1	1	1	1	1	1	1	1	1	1	1	1
140	0	0	0	1	1	1	1	1	0	0	0	1	1	0	1	1	0	1	0	999	0	0	0	1	1	0	0	0	0	0	1	1
