# Supplementary figures and images for: AFLP analysis reveals high genetic diversity but low population structure in Coccidioides posadasii isolates from Mexico and Argentina
Source: BMC Infect Dis. 2013 Sep 3;13:411. doi: 10.1186/1471-2334-13-411 (PMC3766708; doi:10.1186/1471-2334-13-411)

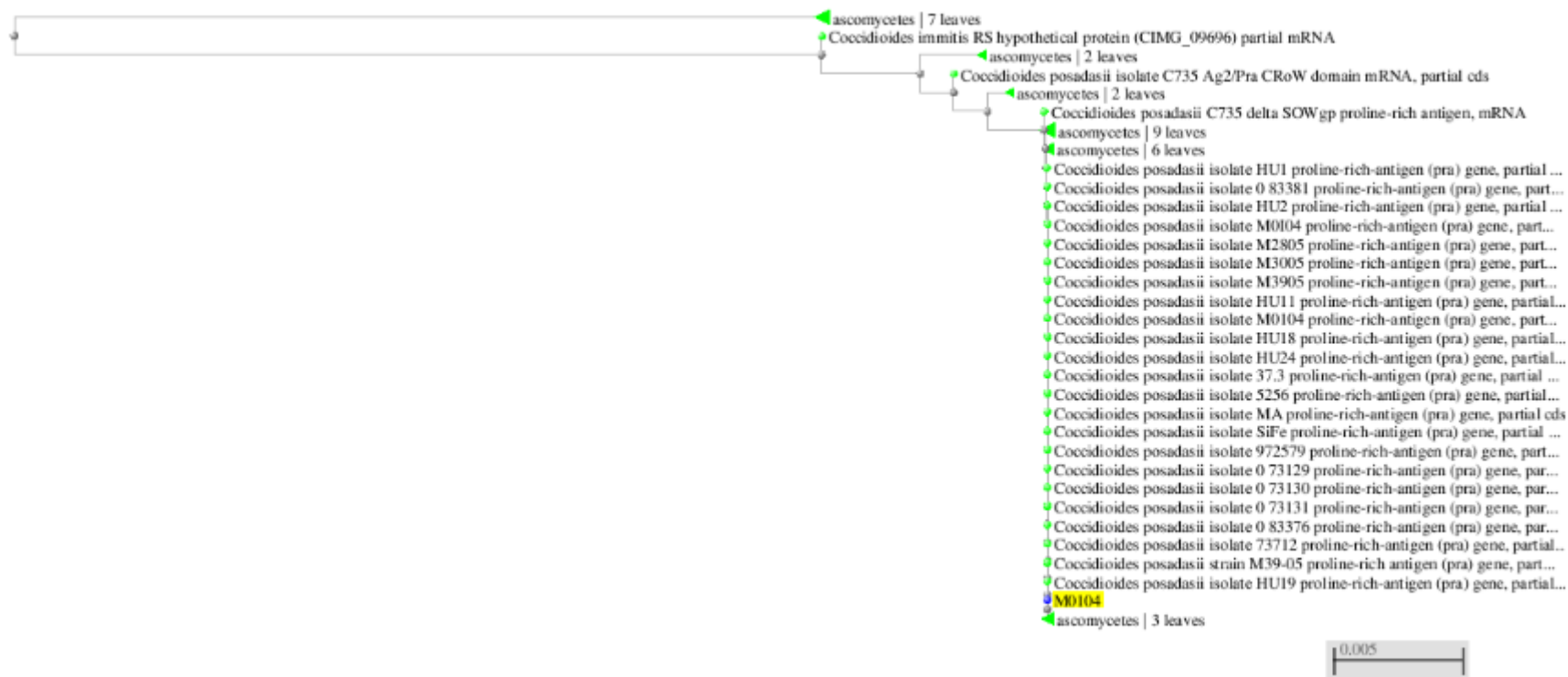

**Additional file 3.** Phylogenetic inference analysis using partial sequences of the Ag2/PRA gene.

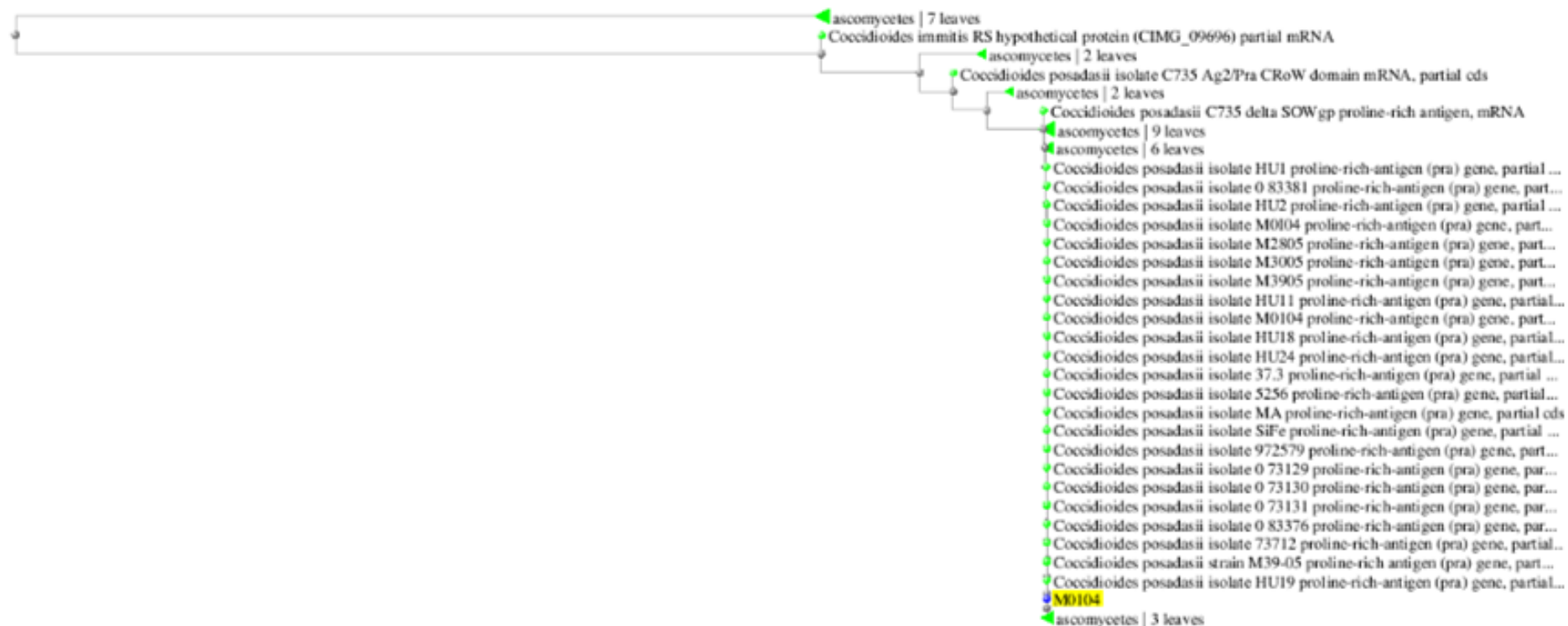

0.005

Supplement: Additional file 3 — The 32 isolates of Coccidioides spp. from MX and AR were identified as C. posadasii trough of the phylogenetic inference analysis. [file 1471-2334-13-411-S3.pdf]
